# Supplementary material for: A systematic pipeline for classifying bacterial operons reveals the evolutionary landscape of biofilm machineries
Source: PLoS Comput Biol. 2020 Apr 1;16(4):e1007721. doi: 10.1371/journal.pcbi.1007721 (PMC7112194; doi:10.1371/journal.pcbi.1007721)
Supplement: S1 Fig — The number of bacterial genomes with different combinations of predicted EPS operons, further represented with their distribution (% bacterial genomes) across different lifestyles and environmental niches. (PDF) [file pcbi.1007721.s001.pdf]

		2	1	20	5	2	10	83	7	1	30	274	101	189
	Acetylated Cellulose	■	■							■				
	Alginate	■	■	■	■	■					■			
	Cellulose						■	■				■		
	Pel			■		■	■		■				■	
	PNAG	■			■	■		■	■					■
	**Total Species Genomes**	**2**	**1**	**20**	**5**	**2**	**10**	**83**	**7**	**1**	**30**	**274**	**101**	**189**
	Lifestyle: Non-Pathogen	100		25	100	100	70	33	86		83	56	63	43
	Lifestyle: Pathogen		100	75			20	66		100	17	38	19	56
	Lifestyle: Unknown						10	1	14			6	18	1
Host-Associated	Niche: Host-Human			55				55			7	30	16	45
Host-Associated	Niche: Host-Other			5				4		100	7	6	6	9
Host-Associated	Niche: Microbiome							1			3	5	1	2
Host-Associated	Niche: Plant	50	100		20		20	12			10	11	9	6
Host-Associated	Niche: Rhizosphere			5	60	100	10	5			10	11	7	3
Environmental /Other	Niche: Food											3	2	3
Environmental /Other	Niche: Freshwater							2			10	3	2	2
Environmental /Other	Niche: Hot spring						10	1				1	4	3
Environmental /Other	Niche: Industrial	50		5				1	14		10	5	10	4
Environmental /Other	Niche: Lab derived			25				13			10	2	2	5
Environmental /Other	Niche: Marine sediment			5					29		7	3	9	6
Environmental /Other	Niche: Seawater						20	1				5	3	1
Environmental /Other	Niche: Soil				20		30	2	43		27	8	12	11
	Niche: Unknown						10	1	14			6	18	1

% of Species Found (Percentile)

100%

10%
