# Supplementary figures and images for: A systematic pipeline for classifying bacterial operons reveals the evolutionary landscape of biofilm machineries
Source: PLoS Comput Biol. 2020 Apr 1;16(4):e1007721. doi: 10.1371/journal.pcbi.1007721 (PMC7112194; doi:10.1371/journal.pcbi.1007721)

## Phylogenetic Diversity of Predicted EPS Operons

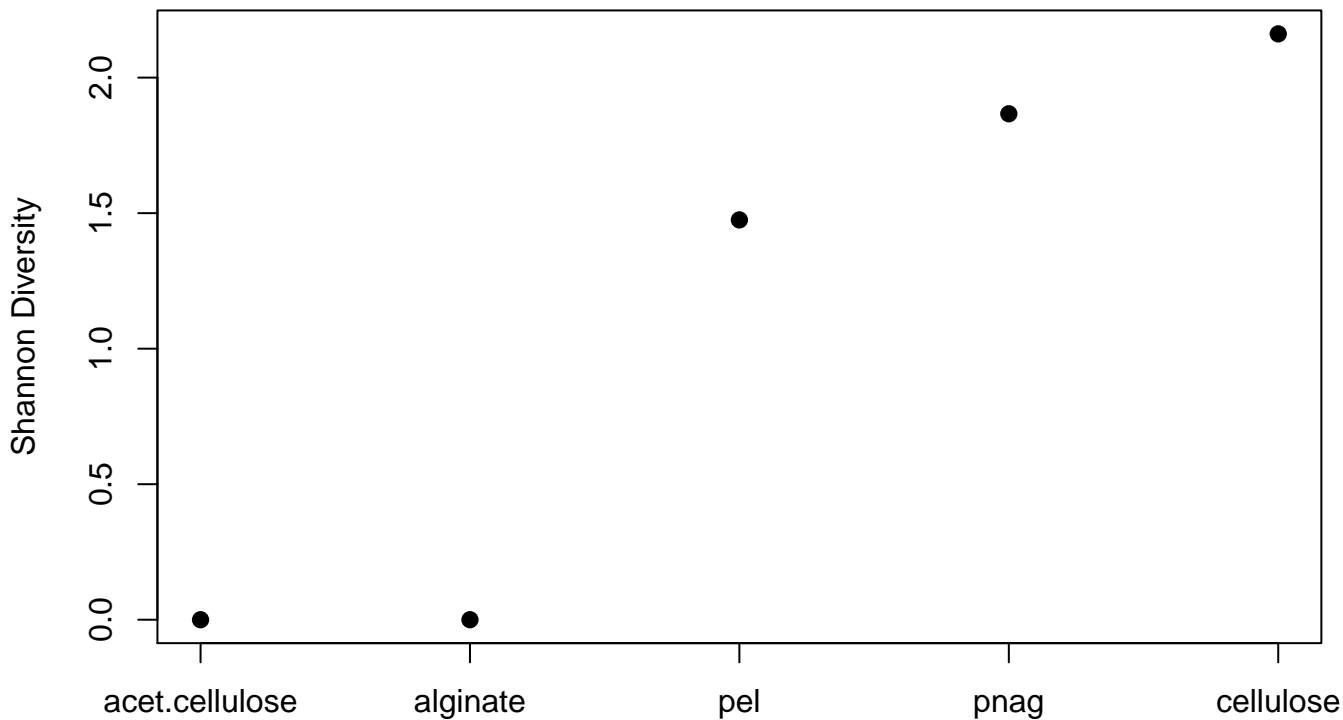

Supplement: S2 Fig — (PDF) [file pcbi.1007721.s002.pdf]

### Additional Loci Identified Through Iterative HMM Searches (HMM e-value <= 1e-5)

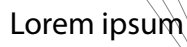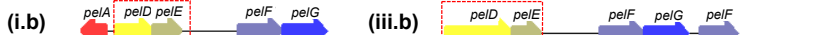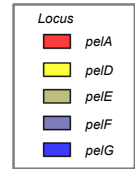

Supplement: S3 Fig — (A) Subnetwork depicting Gram-positive pel operon clades with varying numbers of loci identified as significant matches (e-value < 1e-5) in first-pass (unfilled nodes) and iterative HMM searches (grey nodes). Selected examples shown: (i) PelA-PelFG sequences identified by first-pass HMM hits; (i.b) Iterative HMM searches identifying additional pelA loci in B. cereus ATCC 10987, a known pellicle producing Gram-positive; (ii) Additional pelD loci identified by iterative HMM; (iii) Gram-positive pel operons with only pelF and pelG loci identified. (B) Operon organizations of selected examples of Gram-positive pel operons (corresponding highlighted in panel A) with additional highly divergent loci identified (red boxes: hits above HMM e-value threshold of 1e-5). (PDF) [file pcbi.1007721.s003.pdf]

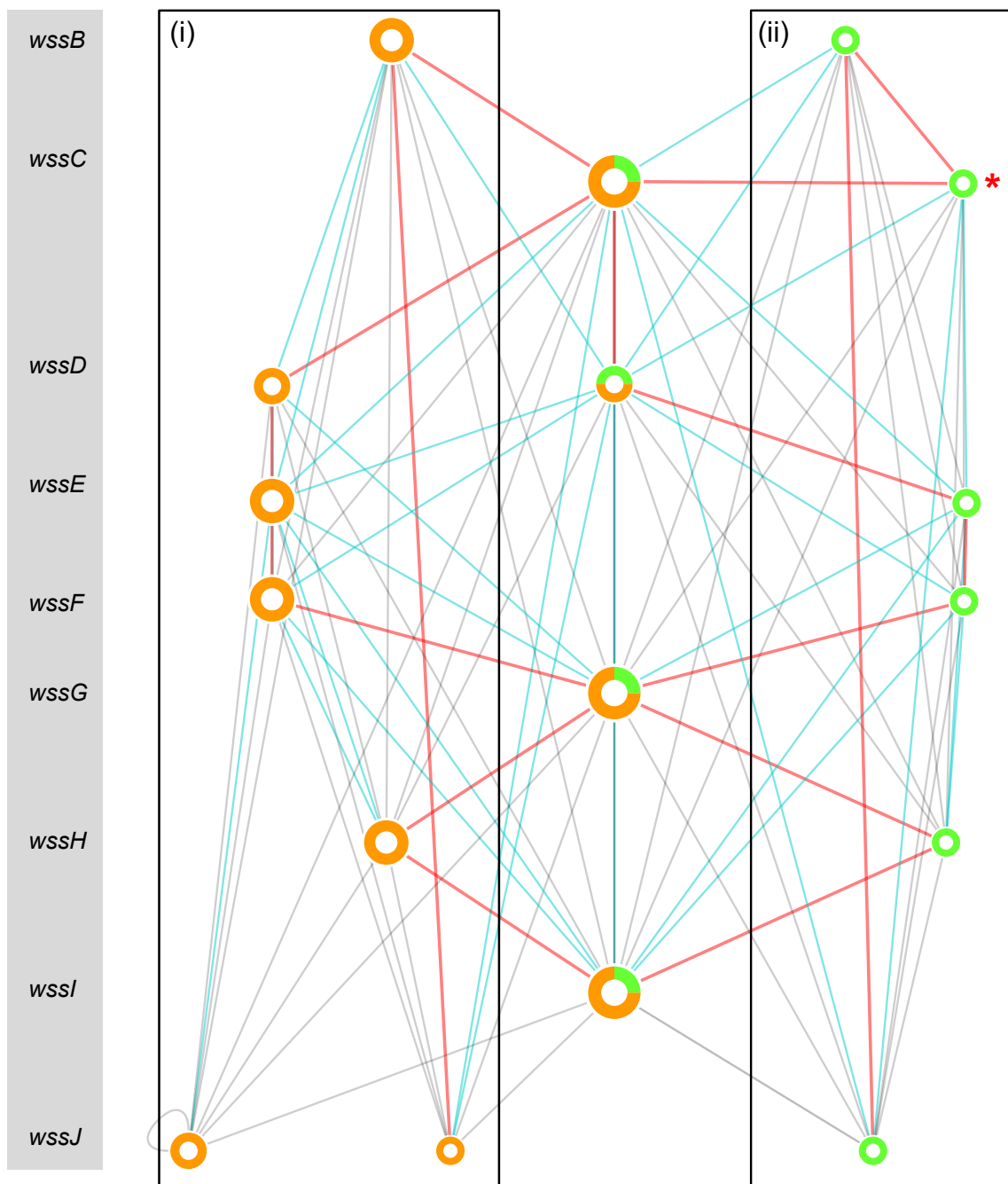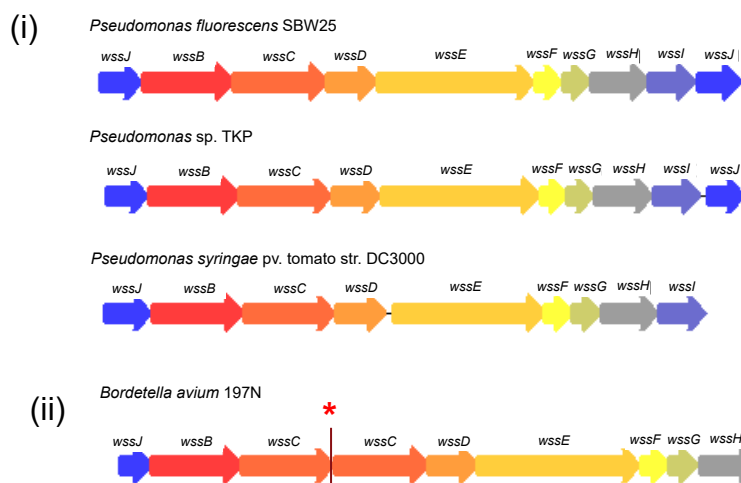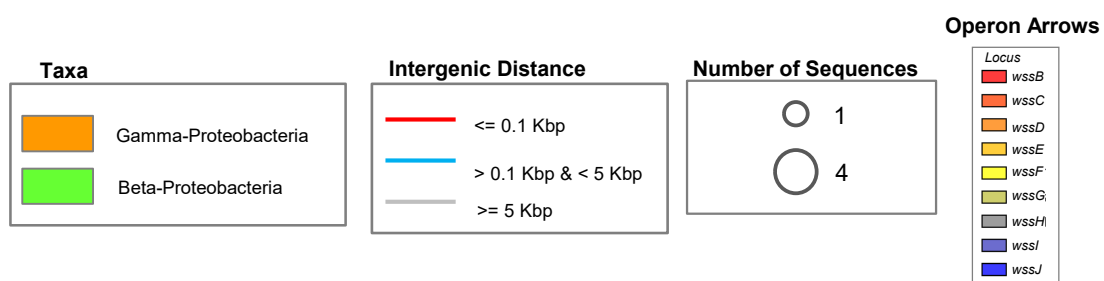

Supplement: S6 Fig — Phylogenetically clustered operon loci are arranged according to the canonical acetylated cellulose operon ordering indicated by the grey sidebar. Inset panels identify three acetylated cellulose operons identified in Pseudomonas spp. (i) and a single Bordetella avium genome possessing a duplicated polysaccharide co-polymerase wssC locus (ii—indicated by red asterisk). Node size indicates the relative number of sequences per phylogenetic cluster; node colouring represents the taxonomic distribution of loci for a given cluster; edges connect clusters which co-occur in the same genome(s); edge colour indicates the genomic-proximity of loci clusters. (PDF) [file pcbi.1007721.s006.pdf]

**A**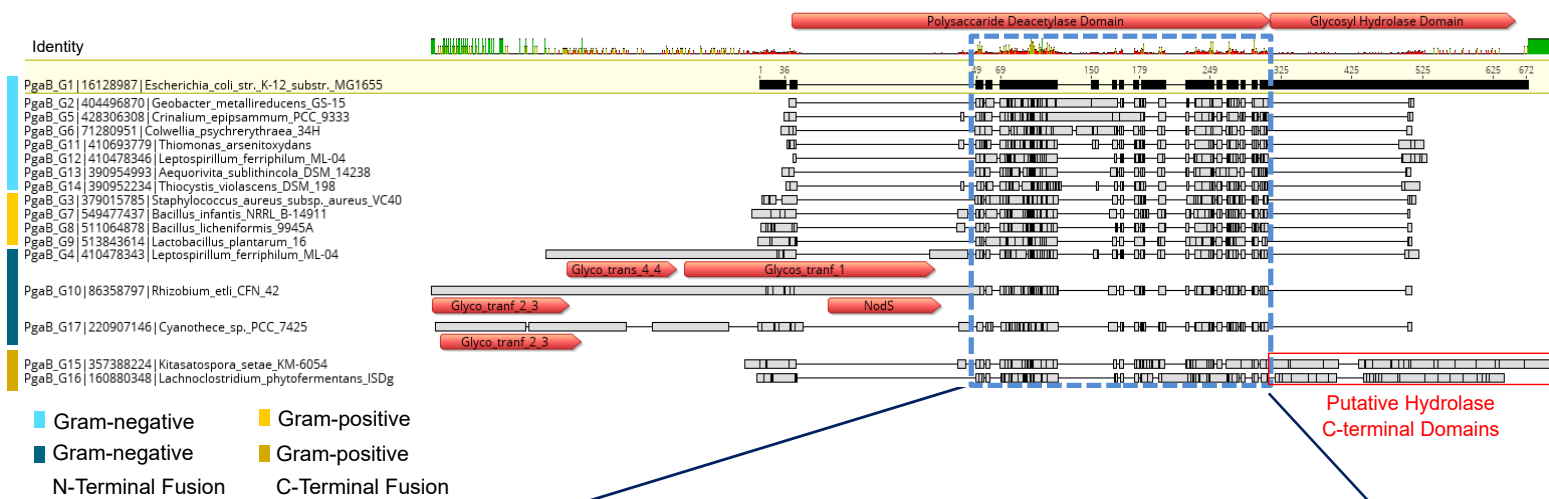**B**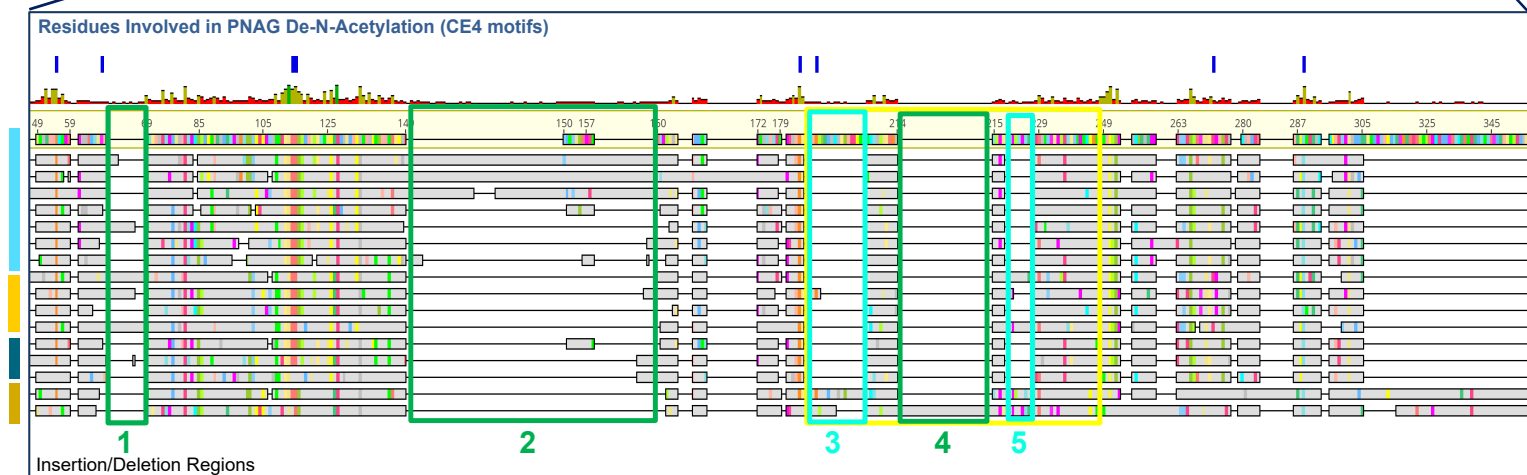**C**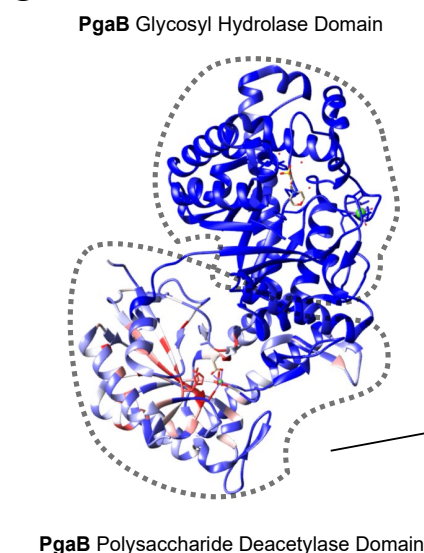**D**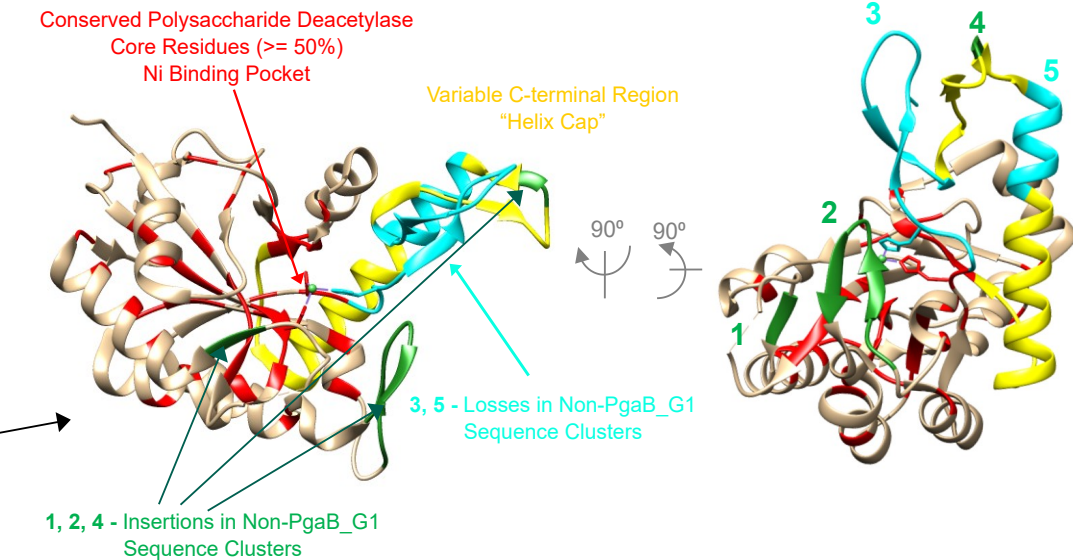**E**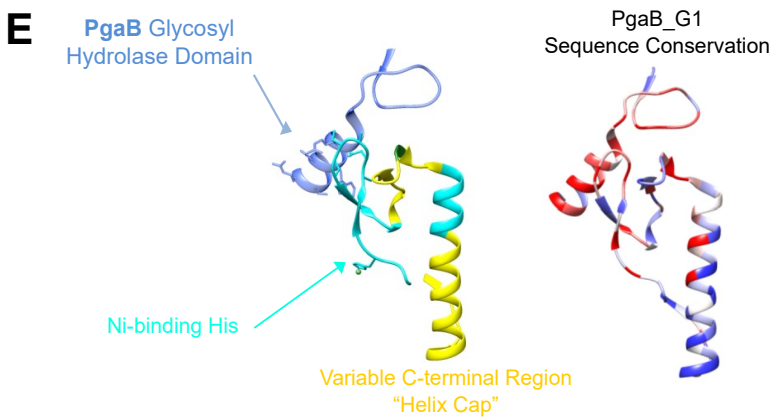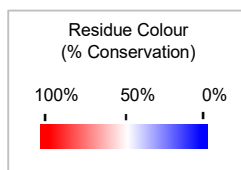

Supplement: S8 Fig — A)—Multiple sequence alignment of representative sequences comprising all PgaB phylogenetic clusters. Global sequence conservation compared against E. coli MG1655 K12 PgaB, phylogenetic cluster PgaB_G1, indicates presence of polysaccharide deacetylase domain (blue box) but an absence of glycosyl-hydrolase domain in non-PgaB_G1 sequences. Red arrows indicate phylogenetic group specific N-terminal domain fusions predicted by PFAM searches; C-terminal domain fusions identified (red box) as putative hydrolase domains from BLAST searches. B)—A close up view of sequence conservation of PgaB polysaccharide deacetylase domains with indel events highlighted: green boxes indicate insertions identified in non PgaB_G1 sequences; teal boxes indicate insertions in PgaB_G1 sequence residing in the C-terminal alpha-helix cap (yellow box). C–Crystal structure of E. coli PgaB (4F9D) indicating conservation of the deacetylase domain catalytic core. D–Deacetylase domain with indel regions indicated according to the colour scheme described for panel B. E–C-terminal alpha helical cap region of the PgaB deacetylase domain indicating insertions of the PgaB_G1 region that are spatially proximal to an N-terminal region of the hydrolase domain (light purple); comparison of the same regions with PgaB_G1 sequence conservation indicated. Multiple sequence alignment was visualized generated using Geneious 10.2.2 (http://www.geneious.com), protein structure was visualized using Chimera 1.11.2 [106]. (PDF) [file pcbi.1007721.s008.pdf]
