# Supplementary material for: A systematic pipeline for classifying bacterial operons reveals the evolutionary landscape of biofilm machineries
Source: PLoS Comput Biol. 2020 Apr 1;16(4):e1007721. doi: 10.1371/journal.pcbi.1007721 (PMC7112194; doi:10.1371/journal.pcbi.1007721)
Supplement: S4 Fig — Phylogenetically clustered operon loci are arranged according to the canonical pga operon ordering indicated by the grey sidebar. Inset boxes depict selected examples of pga operon clades distinguished by evolutionary events: i) Divergence of pgaD corresponding to related enterobacterial species including pathogen-specific losses of pgaA and pgaB loci critical for PNAG export; ii) Operon duplications occurring in aquatic niche dwelling bacteria, including a partial duplication of the pga operon specific to the opportunistic pathogen Acinetobacter baumannii spp. and a whole operon duplication identified in Methylovora versatilis; iii) A unique pga operon organization among environmental bacteria lacking a pgaD locus; iv) Gram-positive ica operons (annotated by their HMM hits to corresponding Gram-negative pga loci) with divergent icaB loci, resulting from novel domain acquisitions (iv.b and iv.c); v) A novel pga derived operon resulting from multiple tandem duplications of the pgaC polysaccharide synthase and lack of detectable pgaA outer membrane pore and pgaD. Node size indicates the relative number of sequences per phylogenetic cluster; node colouring represents the taxonomic distribution of loci for a given cluster; edges connect clusters which co-occur in the same genome(s); edge colour indicates the genomic-proximity of loci clusters. (PDF) [file pcbi.1007721.s004.pdf]

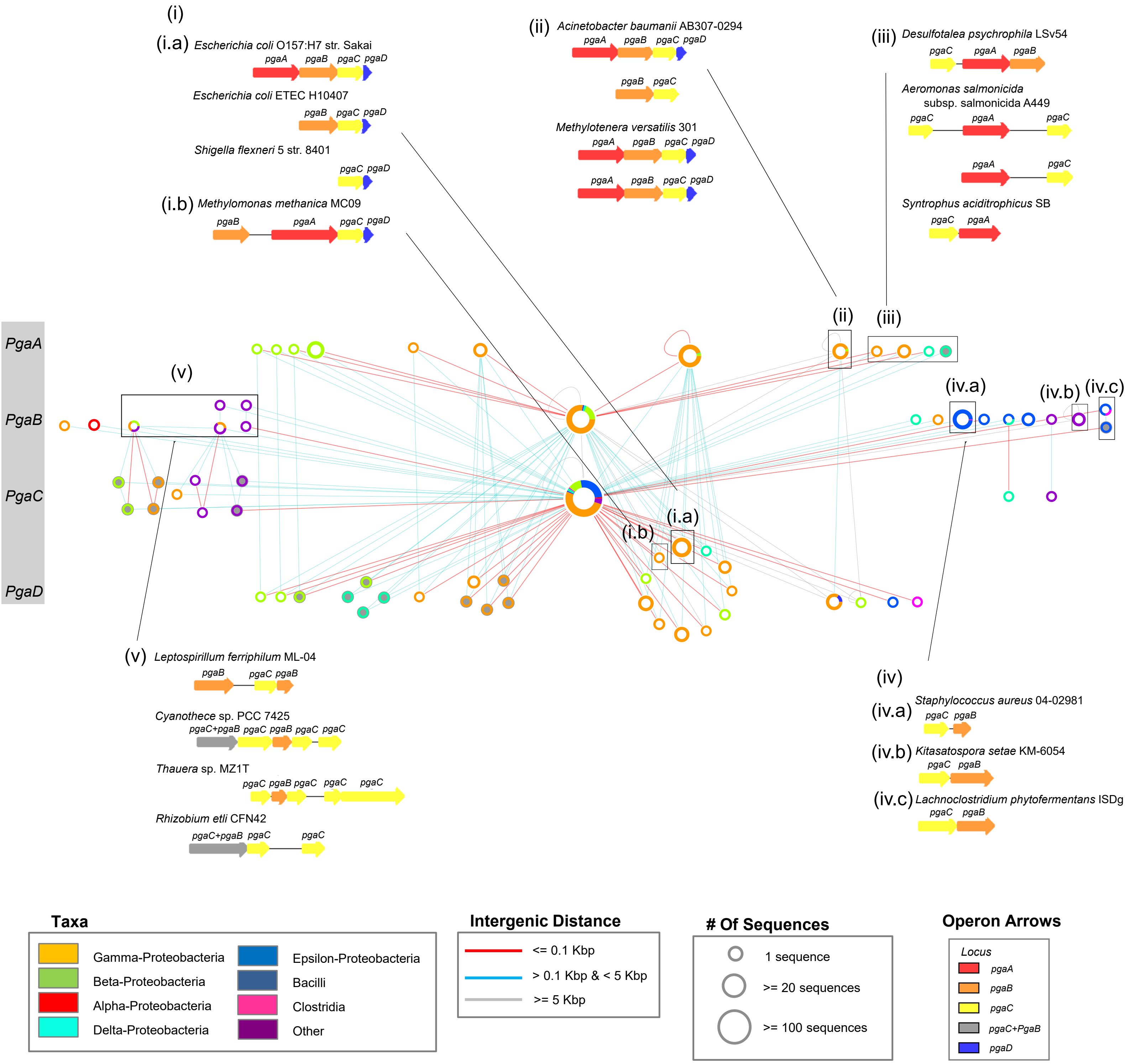
(i)
(i.a) Escherichia coli O157:H7 str. Sakai
pgaA pgaB pgaC pgaD
Escherichia coli ETEC H10407
pgaB pgaC pgaD
Shigella flexneri 5 str. 8401
pgaC pgaD
(i.b) Methylomonas methanica MC09
pgaB pgaA pgaC pgaD
(ii) Acinetobacter baumanii AB307-0294
pgaA pgaB pgaC pgaD
pgaB pgaC
Methylotenera versatilis 301
pgaA pgaB pgaC pgaD
pgaA pgaB pgaC pgaD
(iii) Desulfotalea psychrophila LSv54
pgaC pgaA pgaB
Aeromonas salmonicida subsp. salmonicida A449
pgaC pgaA pgaC
pgaA pgaC
Syntrophus aciditrophicus SB
pgaC pgaA
PgaA
PgaB
PgaC
PgaD
(v)
(ii)
(iii)
(iv.a)
(iv.b)
(iv.c)
(i.a)
(i.b)
(v) Leptospirillum ferriphilum ML-04
pgaB pgaC pgaB
Cyanothece sp. PCC 7425
pgaC+pgaB pgaC pgaB pgaC pgaC
Thauera sp. MZ1T
pgaC pgaB pgaC pgaC pgaC
Rhizobium etli CFN42
pgaC+pgaB pgaC pgaC
(iv)
(iv.a) Staphylococcus aureus 04-02981
pgaC pgaB
(iv.b) Kitasatospora setae KM-6054
pgaC pgaB
(iv.c) Lachnoclostridium phytofermentans ISDg
pgaC pgaB
Taxa
Gamma-Proteobacteria
Beta-Proteobacteria
Alpha-Proteobacteria
Delta-Proteobacteria
Epsilon-Proteobacteria
Bacilli
Clostridia
Other
Intergenic Distance
<= 0.1 Kbp
> 0.1 Kbp & < 5 Kbp
>= 5 Kbp
Of Sequences
1 sequence
>= 20 sequences
>= 100 sequences
Operon Arrows
Locus
pgaA
pgaB
pgaC
pgaC+PgaB
pgaD
