# Supplementary material for: A systematic pipeline for classifying bacterial operons reveals the evolutionary landscape of biofilm machineries
Source: PLoS Comput Biol. 2020 Apr 1;16(4):e1007721. doi: 10.1371/journal.pcbi.1007721 (PMC7112194; doi:10.1371/journal.pcbi.1007721)
Supplement: S5 Fig — Phylogenetically clustered operon loci are arranged according to the canonical alginate operon ordering indicated by the grey sidebar. Inset boxes depict selected examples of alginate operon clades distinguished by evolutionary events: Inset boxes depict selected examples of alginate operon clades distinguished by evolutionary events: i) Canonical alginate operon organization with a partial operon duplication event identified in Pseudomonas resinovorans 136 resulting in the loss of alginate acetylation machinery (ib–indicated by A*); ii) A distinct alginate operon clade (ii.a-c) identified by rearrangement of acetylation machinery (indicated by B*) as well as HGT events with canonical alginate operon possessing species; iii) Atypical alginate operons involving loss of outer membrane transport loci or portions of acetylation machinery in deep sea dwelling bacteria. Node size indicates the relative number of sequences per phylogenetic cluster; node colouring represents the taxonomic distribution of loci for a given cluster; edges connect clusters which co-occur in the same genome(s); edge colour indicates the genomic-proximity of loci clusters. (PDF) [file pcbi.1007721.s005.pdf]

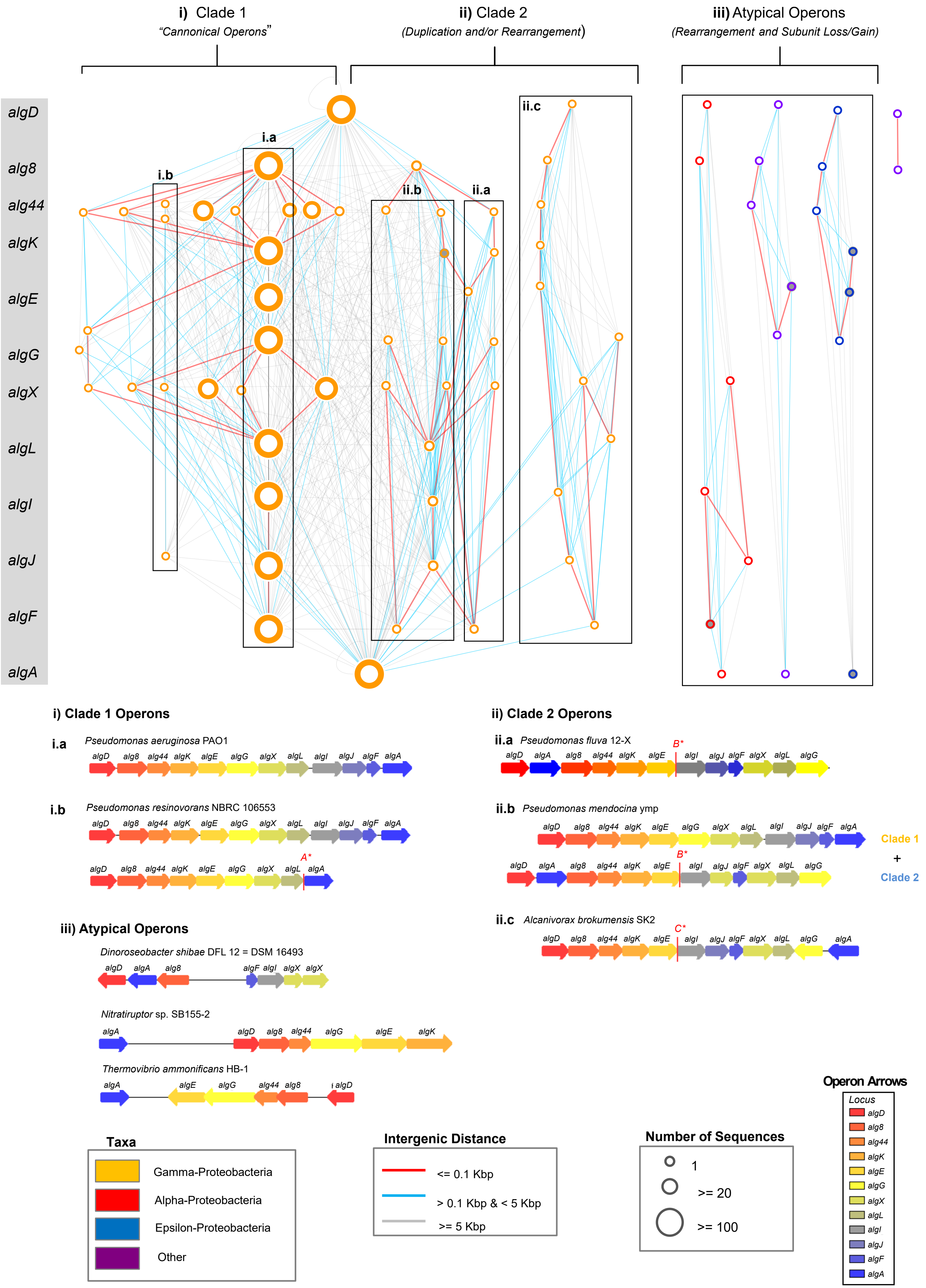
i) Clade 1
"Cannonical Operons"
ii) Clade 2
(Duplication and/or Rearrangement)
iii) Atypical Operons
(Rearrangement and Subunit Loss/Gain)
algD
alg8
alg44
algK
algE
algG
algX
algL
algI
algJ
algF
algA
i.a
i.b
ii.b
ii.a
ii.c
i) Clade 1 Operons
i.a
Pseudomonas aeruginosa PAO1
algD alg8 alg44 algK algE algG algX algL algI algJ algF algA
i.b
Pseudomonas resinovorans NBRC 106553
algD alg8 alg44 algK algE algG algX algL algI algJ algF algA
A*
algD alg8 alg44 algK algE algG algX algL algA
ii) Clade 2 Operons
ii.a
Pseudomonas fluva 12-X
B*
algD algA alg8 alg44 algK algE algI algJ algF algX algL algG
ii.b
Pseudomonas mendocina ymp
algD alg8 alg44 algK algE algG algX algL algI algJ algF algA
Clade 1
+
Clade 2
B*
algD algA alg8 alg44 algK algE algI algJ algF algX algL algG
ii.c
Alcanivorax brokumensis SK2
C*
algD alg8 alg44 algK algE algI algJ algF algX algL algG algA
iii) Atypical Operons
Dinoroseobacter shibae DFL 12 = DSM 16493
algD algA alg8 algF algI algX algX
Nitratiruptor sp. SB155-2
algA algD alg8 alg44 algG algE algK
Thermovibrio ammonificans HB-1
algA algE algG alg44 alg8 algD
Taxa
Gamma-Proteobacteria
Alpha-Proteobacteria
Epsilon-Proteobacteria
Other
Intergenic Distance
<= 0.1 Kbp
> 0.1 Kbp & < 5 Kbp
>= 5 Kbp
Number of Sequences
1
>= 20
>= 100
Operon Arrows
Locus
algD
alg8
alg44
algK
algE
algG
algX
algL
algI
algJ
algF
algA
