# Supplementary material for: A systematic pipeline for classifying bacterial operons reveals the evolutionary landscape of biofilm machineries
Source: PLoS Comput Biol. 2020 Apr 1;16(4):e1007721. doi: 10.1371/journal.pcbi.1007721 (PMC7112194; doi:10.1371/journal.pcbi.1007721)
Supplement: S7 Fig — Top panel—Sequence conservation was mapped onto the cellulose synthase complex, BcsA-BcsB (4HG6 –Rhodobacter sphaeroides ATCC 17025) comprising sequences from eight species representing distinct cellulose operon clades (Fig 4(i)–4(iv)). Lower panels—structural and multiple sequence alignments indicate a high degree of conservation corresponding to BcsA glycosyl hydrolase catalytic core domain and regions of the cellulose translocation channel (i) and UDP binding sites of the BcsA PilZ domain (ii). In Contrast, low overall sequence conservation is found among the carbohydrate binding and ferredoxin domains (CBD1-2, and FD1-2) of BcsB sequences, except the highly conserved cellulose binding site residing in CBD-2 (iii). The translocated cellulose polymer is indicated in green. BcsA domains identified using PFAM predictions for the R. sphaeroides reference sequence, BcsB domains were assigned according to [45]. Multiple sequence alignment was visualized generated using Geneious 10.2.2 (http://www.geneious.com), protein structure was visualized using Chimera 1.11.2 [106]. (PDF) [file pcbi.1007721.s007.pdf]

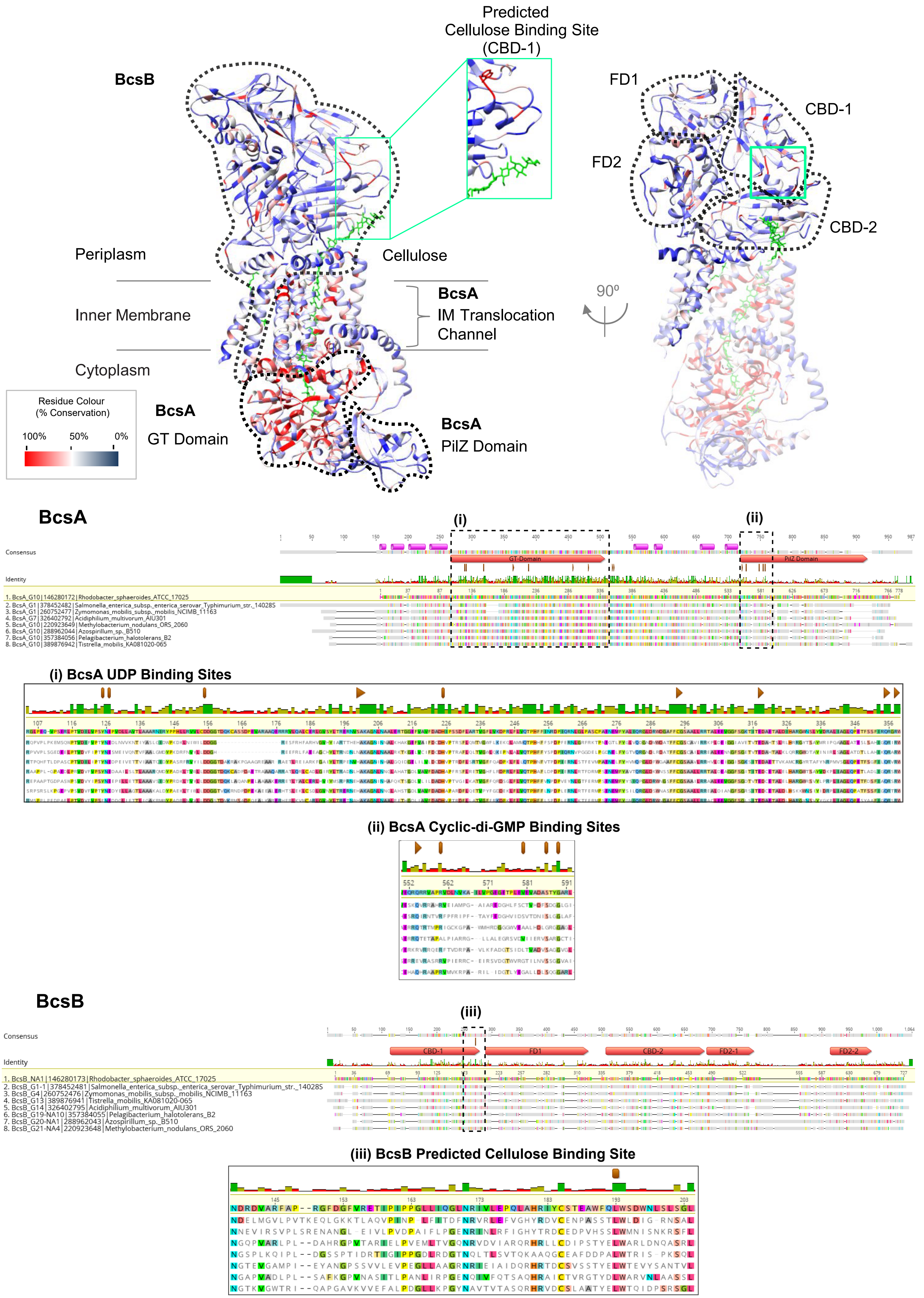
Predicted
Cellulose Binding Site
(CBD-1)
BcsB
FD1
CBD-1
FD2
CBD-2
Periplasm
Cellulose
BcsA
IM Translocation
Channel
Inner Membrane
90º
Cytoplasm
Residue Colour
(% Conservation)
100%
50%
0%
BcsA
GT Domain
BcsA
PilZ Domain
BcsA
(i)
(ii)
Consensus
Identity
GT-Domain
PilZ Domain
1. BcsA_G10|146280172|Rhodobacter_sphaeroides_ATCC_17025
2. BcsA_G1|378452482|Salmonella_enterica_subsp._enterica_serovar_Typhimurium_str._14028S
3. BcsA_G1|260752477|Zymomonas_mobilis_subsp._mobilis_NCIMB_11163
4. BcsA_G7|326402792|Acidiphilium_multivorum_AIU301
5. BcsA_G10|220923649|Methylobacterium_nodulans_ORS_2060
6. BcsA_G10|288962044|Azospirillum_sp._B510
7. BcsA_G10|357384056|Pelagibacterium_halotolerans_B2
8. BcsA_G10|389876942|Tistrella_mobilis_KA081020-065
(i) BcsA UDP Binding Sites
(ii) BcsA Cyclic-di-GMP Binding Sites
BcsB
(iii)
Consensus
Identity
CBD-1
FD1
CBD-2
FD2-1
FD2-2
1. BcsB_NA1|146280173|Rhodobacter_sphaeroides_ATCC_17025
2. BcsB_G1-1|378452481|Salmonella_enterica_subsp._enterica_serovar_Typhimurium_str._14028S
3. BcsB_G4|260752476|Zymomonas_mobilis_subsp._mobilis_NCIMB_11163
4. BcsB_G13|389876941|Tistrella_mobilis_KA081020-065
5. BcsB_G14|326402795|Acidiphilium_multivorum_AIU301
6. BcsB_G19-NA10|357384055|Pelagibacterium_halotolerans_B2
7. BcsB_G20-NA1|288962043|Azospirillum_sp._B510
8. BcsB_G21-NA4|220923648|Methylobacterium_nodulans_ORS_2060
(iii) BcsB Predicted Cellulose Binding Site
