# Supplementary material for: A systematic pipeline for classifying bacterial operons reveals the evolutionary landscape of biofilm machineries
Source: PLoS Comput Biol. 2020 Apr 1;16(4):e1007721. doi: 10.1371/journal.pcbi.1007721 (PMC7112194; doi:10.1371/journal.pcbi.1007721)
Supplement: Attachment — Submitted filename: Response.docx [file pcbi.1007721.s017.docx]

*Editors Comments:
The reviewers all found the manuscript to be a potentially important contribution. They make a number of suggestions for improvement. One reviewer remarks that the novelty of your study resides more in the results than development of a new method. This should be addressed - indeed, if you feel it is appropriate you can re-orient the narrative of the paper. All reviewers raise a number of technical issues which should be addressed with revisions or clarification. The reviewers also point out the need for the senior authors to check the manuscript for language errors. In fact, all authors should carefully read and edit the text.*

We would like to thank you and the reviewers for your highly encouraging comments. As suggested, we provide more details on previous applications of operon-based investigations and expand more on the potential novelty of this study, both in terms of methods and from the findings produced by our study. As noted below, we also address each of the technical concerns that were previously raised and have carefully edited the text to improve the language. We believe addressing these concerns has significantly strengthened this paper.

*---*

*Reviewer's Comments

Reviewer #1: The paper by Bundalovic-Torma and colleagues presents a comparative analysis of the operons that encode the enzymes synthesizing five exopolysaccharides: cellulose, acetyl-cellulose, poly-N-acetylglucosamine, Pel (a polymer of mannose, rhamnose, and glucose residues), and alginate (mannuronate/guluronate polymer).
The operons are clustered and compared on the basis of their gene content, phylogenetic distribution, and the likely evolutionary history (gene rearrangement, duplication, loss, fusion,and horizontal gene transfer). This is an interesting and potentially useful work.*

We would like to thank the reviewer for their support of our work and the constructive comments provided.

*However, as could be expected for such an ambitious project, a number of important issues have been either glossed over or relegated to the supplementary files. The paper would benefit from addressing the following points*.

*Major comments.*

*1. The paper is entitled "A systematic pipeline for classifying bacterial operons...". Unfortunately, I do not see a clear description of any pipeline. An explanatory figure or a flow chart would be helpful. It would also be useful to highlight which steps of the pipeline result in which results (predictions, discoveries).*

We now provide an **additional panel in Figure 1** highlighting the overall schema we have used for the systematic prediction and analysis of bacterial operons. At the same time, details of the phylogenetic clustering steps and generation of genomic-proximity networks are presented in **Figure 2.**

These steps are:

1. The construction of EPS locus HMM models
2. HMM searches of loci against fully sequenced bacterial genomes.
3. Prediction of putative operon loci through genomic-proximity reconstructions.
4. Classification of operon clades through phylogenetic tree construction and evolutionary-distance based sequence clustering.
5. Genomic-proximity network visualization of operon clades to aid the large-scale investigation and discovery of distinct operon organizations across diverse bacterial phyla.

We further note (**Page 7, Lines 165-170**) that the majority of our EPS operon predictions are from bacteria that have not been previously characterized. We have endeavored to emphasize the additional value of our work as a resource for future discovery of potential EPS producing bacteria, as demonstrated in our accompanying validation manuscript.

*2. Given the numerous errors in language (see below for examples), which is unexpected for a Toronto-based team, it is essential that the senior author(s) read and checked the entire text.*

We appreciate the attention of the reviewer to our manuscript, and have carefully edited the manuscript for language errors that were previously missed.

*2. This ms fails to mention the first publication on the use of operon structure to predict functional coupling: Overbeek et al. 1999, PNAS 96 (6) 2896-2901,* [*https://www.pnas.org/content/96/6/2896*](https://urldefense.proofpoint.com/v2/url?u=https-3A__www.pnas.org_content_96_6_2896&d=DwMFaQ&c=Sj806OTFwmuG2UO1EEDr-2uZRzm2EPz39TfVBG2Km-o&r=QD7AAukDn_FTFKywmUAPF2xmvPH1T38zLXt2ePQBhjg&m=ddgSzhewK6BAXJccuTr8F54cv9PhwyEvtwtV_qQMCDo&s=66B72mS8h9K5h6E2O87mEp_z2ValcNH-rMlHTX6AjVQ&e=)*. This paper needs to be properly acknowledged.*

We have incorporated the reference suggested by the reviewer in the introduction (**Page 4, line 73**).

*3. The authors do not explain why they have limited their description of the cellulose synthase operon to just four genes, one of which, bcsZ, actually encodes an inhibitor of cellulose biosynthesis (Ahmad et al. 2016, Microb Cell Fact. 15:177. PMID: 27756305). Traditionally, the reference cellulose synthase operon was the best-studied bcsABCD from Gluconobacter xylinus. As detailed in a recent review (ref. 25), the bcs operon in E. coli and Salmonella consists of at least 6 genes, with three more involved in cellulose modification by phosphoethanolamine (Thongsomboon et al. 2018, Science 359:334-338, PMID: 30232265). Ignoring these extra genes and the additional modification of cellulose simplifies the calculations but results in a biased presentation of the diversity of cellulose synthase operons. At the very least the existence of the extra genes and the associated modification must be acknowledged and the reasons for trimming the gene set carefully discussed.*

A major motivation for this study was to provide an agnostic approach for the characterization of bacterial operon encoded machineries. We selected synthase-dependent EPS systems to illustrate the utility of our approach. In general, EPS systems have been characterized in a relatively limited set of taxa. Through our systematic analyses our aim was to provide insights into how these systems have evolved and may have contributed to the adaptation of their host bacteria to different environments. We now provide additional text detailing this motivation (**Page 7, lines 146-151**).

We nonetheless agree with the reviewer that many cellulose biosynthesis operons contain more than the four representative genes chosen for our analysis (*bcsA*, *bcsB*, *bcsC*, and *bcsZ*). We limited our query to *bcsA*, *bcsB*, *bcsC*, and *bcsZ* based on the observation that these are the most abundant genes when comparing all identified cellulose biosynthesis operons (based on Romling and Galperin 2015, Trends Microbiol. 23: 545 PMID: 26077867). Since, at the very least, a functional cellulose biosynthesis locus should contain the synthase genes *bcsA* and *bcsB*, we are confident that our analysis has resulted in few, if any, false-negative predictions.

We have modified the text to acknowledge the existence of additional *bcs* genes and further detail our rationale for focusing on the four selected genes in the main text (**Page 12 lines 273-281**). Regarding the observations outlined by Thongsomboon *et al*. regarding the production of phosphoethanolamine-modified cellulose, we note that our analyses identified cellulose operons in *E. coli* strains UTI89 and W3110 (AR3110) and *S. typhimurium* strain ATCC 14028 (IR715). Examination of the genomic context surrounding the core *bcsA*, *bcsB*, *bcsC*, and/or *bcsZ* loci identified in this work would undoubtedly reveal additional *bcs* genes. However, given that there are upwards of 25 cellulose biosynthesis-associated genes, many of which are lineage-specific, we opted to focus on the four most common of these.

We also agree with the reviewer that the work of Ahmad *et al*. revealed a role for BcsZ as an inhibitor of biofilm phenotypes in *S.* typhimurium. However, in *Komagataeibacter xylinus*, BcsZ is required for cellulose biosynthesis (Koo et al. 1998 Biosci Biotechnol Biochem 62: 2257 PMID 9972249; Kawano et al 2002 J Biosci Bioeng 94: 275 PMID 16233303; Nakai et al 2013 J Bacteriol 195: 958 PMID 23243308), suggesting that the function of BcsZ is likely species-specific.

*4. In this paper, any deviations from the reference gene set are described in evolutionary terms (gene rearrangement, duplication, acquisition and loss), implicitly assuming that chosen the reference sets are ancestral operons. Since there is no evidence presented for this assumption being correct for any analyzed operon, all these terms must be qualified and the likely pathways of the evolution of the respective operons discussed without making any unnecessary assumptions.*

We have clarified in our manuscript that gene loss, rearrangement, duplication, and acquisition signify a difference in composition and/or organization between a reference and predicted EPS operon and that we do not assume that the reference operon is ancestral representative (**Page 10, lines 223-225**).

*5. It is not clear whether the trimmed operons actually produce the same compound. The example of Pel biosynthesis by B. cereus is very impressive but it could still be an exception.*

We appreciate the reviewer’s comment that our sequence homology searches uncovered the existence of a distantly related and distinctly organized *pel*-like operon in *B. cereus* that could be validated as a Pel producing biosynthetic cluster.

The validation of a *pel* biosynthetic cluster in *B. cereus*, described in the accompanying manuscript which has now been accepted for publication by PLoS Pathogens) is the first and crucial piece of evidence that strongly suggests that divergent Gram-positive *pel* operons, particularly those belonging to the same phylogenetic clade as *B. cereus,* possess the ability to produce the Pel polysaccharide. However, we agree with the reviewer, that without accompanying experimental validation significantly divergent operon loci may not result in the production of an identical EPS, as the case PNAG modification between Gram-negative (*pga)* and Gram-positive (*ica*) operons (Whitfield et al 2015 Front Microbiol 15: 471 PMID 26029200). Text clarifying that identified operons may not result in the production of biofilms has now been added to the main text (**Page 17, lines 386-391**).

*6. Supplemental Table 7 is a very useful product of this work. Any chance it could be made available to the community as a separate resource (e.g. a web site), not getting lost among 15 other supplementary files?*

We thank the reviewer for their positive comment. We have updated our manuscript to note that Supplemental Table 7 (now Supplemental Table 8) as well as the HMM models used in our prediction pipeline are available online from a github repository (<https://github.com/ParkinsonLab/eps_biofilms>) (**Page 31, lines 729-730**).

*Minor comments.
L. 52. "all known synthase dependent bacterial biofilm machineries". Not all, there is also Psl, levan, and phosphoethanolamine cellulose, and probably other biofilm-forming polysaccharides.*

The phosphoethanolamine-modified form of cellulose was not yet identified at the outset of this work, but nevertheless the *bcsABC* components of these loci were uncovered by our search (see comment #3 above) and are grouped together with other cellulose operons. Psl is synthesized by a Wzx/Wzy-dependent pathway and so is not synthase-dependent (Franklin et al 2011 Front Microbiol 22: 167 PMID 21991261). Based on the criteria currently used to define the synthase-dependent mechanism (Whitney & Howell 2013 Trends Microbiol 21: 63 PMID 23117123; Low & Howell 2018 Curr Opin Struct Biol 53: 32 PMID 29843050), levan biosynthesis would not utilize this pathway.

*L. 67. Strictly speaking, the definition of an operon includes co-regulation. Rephrase.*

We have rephrased the wording (**Pages 2-3, lines 47-49 & 70**).

*L. 69. What do you mean by "co-conserved"? Rephrase.*

We have rephrased the wording (**Page 3, line 70**)

*L. 70. Remove "subsequently" (or move to the beginning of the sentence).*

We have made the change as suggested (**Page 4, line 78**).

*L. 71 function isolation -> function in isolation*

We have made the change as suggested (**Page 4, line 78**).

*L. 78-79. "role of evolutionary events on operon structure" Role in or Influence on?*

We have made the change as suggested (**Page 4, line 86**).

*L. 103. "EPS are an important constituent". Rephrase.*

We have made the change as suggested (**Page 6, line 131**).

*L. 117-118. Again, what about the Psl and phosphoethanolamine cellulose?*

Please see comment to L. 52 above.

*L. 138. these finding -> these findings*

We have made the change as suggested (**Page 8, line 172**).

*L. 156. Actinobacteridae and Rubrobacteridae are obsolete names for the classes Actinobacteria and Rubrobacteria, see* [*https://www.ncbi.nlm.nih.gov/Taxonomy/Browser/wwwtax.cgi?id=201174&lvl=1*](https://urldefense.proofpoint.com/v2/url?u=https-3A__www.ncbi.nlm.nih.gov_Taxonomy_Browser_wwwtax.cgi-3Fid-3D201174-26lvl-3D1&d=DwMFaQ&c=Sj806OTFwmuG2UO1EEDr-2uZRzm2EPz39TfVBG2Km-o&r=QD7AAukDn_FTFKywmUAPF2xmvPH1T38zLXt2ePQBhjg&m=ddgSzhewK6BAXJccuTr8F54cv9PhwyEvtwtV_qQMCDo&s=gUk3xw71UE3rIUHQJA20eGm_Dy4q3r_WcVPSuborn90&e=)

We thank the reviewer for their comment. We have updated the associated nomenclature (e.g. **Page 9 line 202**)according to the SILVA database taxonomic procedure (<https://www.arb-silva.de/documentation/silva-taxonomy/>) which relies on phylogenetic-based assignment of taxonomic nomenclature adopted from the Genome Taxonomy Database (Parks et al 2018 Nat Biotechnol 36: 996 PMID 30148503 <https://gtdb.ecogenomic.org/>).

*L. 170. "The processes ... is poorly understood."*

We have made the change as suggested (**Page 9, line 212**).

*L. 179-180. " the resulting number ... were assessed"*

We have made the change as suggested (**Page 10, line 221**).

*L. 180. ordering -> order*

We have made the change as suggested (**Page 10, line 222**).

*L. 338. pathogen enterobacteria -> pathogenic enterobacteria*

We have made the change as suggested (**Page 17, line 398**).

*L. 384 and elsewhere. Acetylated cellulose does not need a dash.*

We have made the change as suggested.

*In the Supplementary Table 6, the "NCBI Genbank Genome ID" column are actually RefSeq genome IDs. RefSeq is different from GenBank, e.g. the RefSeq entry NC_015671 for Cellulomonas [Cellvibrio] gilvus ATCC 13127 corresponds to the GenBank entry CP002665.*

We have made the change as suggested (now **Supplemental Table 7**).

***Reviewer #2:*** *The manuscript entitled « A systematic pipeline for classifying bacterial operons reveals the evolutionary landscape of biofilm machineries » by Bundalovic-Torma and colleagues represent an incredible effort to characterize the composition and evolution of the five major EPS operons known across the bacterial diversity.

The authors should be commended by the amount of work performed and the depth of their analysis and all the details provided. Despite the load of work, the results are presented, in most cases, clearly and the figures in the text (and supplements) provide a lot of interesting and insightful information.

Overall, I think this manuscript is of interest to the biofilm and comparative genomics community, it provides novel results and further understanding the evolution of different EPS operons. The reviewer also appreciated the analysis of the genomic results in light of the biological function each protein has. There are however, a few points that I feel should be address prior to acceptance.*

We would like to thank the reviewer for their highly positive and encouraging remarks.

Major comments
*• Validation of results. My major concern relies on the validation of the EPS predictions. How was this performed? Typically, in a real agnostic way, the database of reference genomes should have been split in two, one should be used for generating the models (HMM profiles and genomic reference organization) and the other one as a validation dataset. Was this done?*

*Further, there is no evidence that the results obtained were systematically contrasted with any published literature, at least a random subset of those.
I would suggest that the authors validate their data using new genomes from NCBI. (They worked on genomes from 2015, and NCBI has most likely doubled the number of genomes since then).*

*I am aware that there is an accompanying manuscript validating one novel EPS in Bacillus, but it would provide robustness to this manuscript if a more thorough literature search would be done and potentially detect false negatives, or maybe even false positives.*

In this analysis, our goal was to provide a comprehensive survey of putative EPS operon systems, for which the criterion of a putative EPS operon is provided in the methods section, “Prediction of EPS Operons” (**Pages 30-31**), in the manuscript. We see the application of this dataset as providing a classification scheme of operon lineages based on their evolutionary relationships, which can be performed in a large-scale and agnostic fashion. A challenge in validation of such analyses is the overall sparsity of experimentally validated EPS operons (true positives), and even sparser availability of species which possess an EPS operon but do not produce a biofilm. For example, the loss of the *pgaAB* loci in pathogenic enteric bacteria *Shigella flexneri* 5 str. 8401, and loss of PNAG operons in *Salmonella enterica* are two examples we are aware, of organisms which have been specifically characterized or commented on as lacking biofilm production capabilities (**Pages 17-18, lines 400-410**).

In light of this reviewers comments we performed a comprehensive literature search and compared our operon predictions against experimentally characterized cellulose, acetylated cellulose, PNAG, pel, and alginate species (**See Supplementary Table 4 and Page 8, lines 187-192**). We note for all characterized species with a fully sequenced genome we were successful in detecting an operon corresponding to the type of EPS produced. Furthermore, for experimentally characterized species lacking a fully sequenced genome, we identify several closely related strains harboring an operon identical to the characterized EPS produced, providing a valuable resource for further experimental validation.

***Note: we can only validate experimentally characterized species if those species are present in the set of genomes we used in our searches (retrieved April 2015). On the other hand, our set of genomes do contain the majority of experimentally confirmed EPS producing species; we therefore expect that reperforming our analyses with a more recent version of the NCBI genomes database, would not substantially change our results.

*• L157-158: Statistical analysis. I do not understand how a t-test could have been done with this data. This data concerns distribution of a trait (EPS) across a character (pathogen-non pathogen). This should be analysed using contingency tables and the appropriate statistical analysis would be a Chi-squared or a Fisher’s test but not a T-test. Further, given that there are multiple EPS operons, this should be then corrected post hoc for multiple observations. Statistical analysis should be redone and claims of link towards pathogenicity should be modified accordingly.*

We thank the reviewer for the comment. We have repeated statistical testing using the chi-square test and updated the manuscript accordingly (**Page 9, lines 193-198**). As tests for pathogen vs. non-pathogen distribution enrichment are performed on the distribution of each EPS operon system separately and repeatedly performed between EPS systems multiple-testing correction is not required.

*• L157-158: In some occasions, pathogenicity is not a well-defined trait. For example, many species are not pathogenic but there can be a reference in which in a given patient they may have caused disease. For example, commensal bacteria can under certain circumstances (immunosuppressed individuals) become facultative pathogens. Were these cases consistently assigned as pathogens or non –pathogen? This should be more explicitly mentioned in the methods sections. Can you explain for example why two very similar S. aureus, “(Sequence type (ST) 59 from an epidemic lineage of community-associated (CA) methicillin-resistant Staphylococcus aureus (MRSA) isolates. Taiwanese CA-MRSA isolates belong to ST59 and can be grouped into 2 )” were classified as pathogenic (SA957) and non-pathogenic (SA40)?*

For each bacterial strain predicted to possess an EPS operon, metadata detailing the isolation source, including the location and year where applicable, were manually curated from the literature by searching for articles citing details of the bacterial strain using both NCBI Pubmed and Google Scholar (**Supplemental Table 8**). We categorized these strains as pathogenic or non-pathogenic based on the literature reference describing the isolation source for the specific strain. For example, the *Staphylococcus aureus* strains SA40 and SA957 are both community-acquired methicillin-resistant clones from the same epidemic ST59 lineage (Chen et al 2013 PLoS ONE 8: e63210 PMID 24039691). However, SA40 was originally isolated from a nasopharyngeal swab of a healthy child and is generally associated with commensal colonization of healthy individuals, while SA957 was isolated from the blood of a patient with severe pneumonia and is commonly associated with disease. This has been linked to the presence of the Panton–Valentine leukocidin (PVL) genes in SA957 and their corresponding absence in SA40 (Chen et al 2013 PLoS ONE 8: e63210 PMID 24039691). Based on this information gathered from literature sources, we categorized SA40 as non-pathogenic and SA957 as pathogenic.

*• Figure 1, L 148-157 I believe it would be more informative to provide the percentage of genomes in which each EPS was found rather than absolute numbers (i.e. as shown in Figure S1). I also believe that for visualization purposes, a figure with a tree of the bacterial diversity and the presence/absence of each EPS could be shown in each clade (or better, the degree prevalence (%) of each EPS on each clade).*

As suggested, percentages are now used in Figure 1 (updated panels B and C), and a cladogram of operon distributions has been added (panel D).

*• The authors present this paper at the beginning as a methodology paper. I think this is an overstatement, and somehow undermines the important results (evolutionary and biological results) presented here. Authors refer to a “novel” method to study the evolution of operons using phylogenetic clustering. I am confused as to what is the precise novelty of this approach. There are a number of papers that have used genomic operon architecture & phylogenetics to study the evolution for instance of secretion systems in bacteria (doi: 10.1371/journal.pgen.1002983, doi: 10.1371/journal.pbio.3000390), as well as stand-alone programs to detect the presence of complex operons in genomes (doi:10.1371/journal.pone.0110726). I would tone down the novelty of the method and focus directly in the evolutionary results.*

We would like to thank the reviewer for the informative references on previously existing phylogenomics-based approaches for the study of bacterial biological machineries. We feel that the novelty of our approach is in its broad application toward the study of bacterial operons in general, particularly as it relies upon an unsupervised method defining evolutionary clustering thresholds for gene families. We further demonstrate its utility by elucidating the evolutionary mechanisms that have given rise to novel operon lineages across phylogenetically diverse species. We have modified our manuscript to highlight these methodological differences to previous approaches and have incorporated the provided references (**Page 5-6, lines 109-123; Page 10, lines 223-225)**.

*Minor comments
• L71 word missing? “do not function in isolation”*

We have made the change as suggested (**Page 4, line 78**).

*• L82 more references could be added for example that investigating the divergence between the outer membrane exporter of bacterial capsules and EPS (*[*https://doi.org/10.1016/B978-0-12-394313-2.00007-X*](https://urldefense.proofpoint.com/v2/url?u=https-3A__doi.org_10.1016_B978-2D0-2D12-2D394313-2D2.00007-2DX&d=DwMFaQ&c=Sj806OTFwmuG2UO1EEDr-2uZRzm2EPz39TfVBG2Km-o&r=QD7AAukDn_FTFKywmUAPF2xmvPH1T38zLXt2ePQBhjg&m=ddgSzhewK6BAXJccuTr8F54cv9PhwyEvtwtV_qQMCDo&s=f2w-a60Fn7Pp9L4IE549xkjCpGST1C_qAnm5mJvGAc4&e=)*, doi: 10.1128/MMBR.00024-08)*

We have added additional references as suggested (**Page 4, lines 90-95**)

*• Figure 1D was confusing at first. Wouldn’t it be simpler to do a more traditional co-occurrence matrix? Or a small interaction network? Or maybe modify the legend. I felt it was somewhat counterintuitive to read the table vertically.*

Owing to the sparsity of operon combinations observed, we felt that both co-occurrence matrix and network visualization would not adequately highlight the specific patterns of operon combinations observed. We have modified Figure 1D (now Figure 1E) for easier visual interpretation as an upset plot.

*• L199-200 It would be of use for the molecular microbiologists to add to the methods the precise genes/proteins used to generate the multiple sequence alignment, and why were those genes chosen. Were those core genes for each operon? Those described in the literature as essential for the biosynthesis? For instance, concerning cellulose operon, in several E. coli natural isolates, the operon is a divergent operon with bcsABZC on one side and bcsEFG on the other side (both of which are essential for cellulose production, doi:10.1111/j.1365-2958.2009.06678.x).*

We have updated our manuscript to provide further detail on the selection of EPS operon loci for HMM construction and operon prediction, and in particular for the selection of cellulose loci (**Page 12 lines 273-281**). The selection of *bcsABZC* were selected based on their broad distribution across known cellulose producing bacteria, while *bcsEFGR* are not as well conserved across proteobacteria, e.g. *Pseudomonas fluorescens* SBW25*, Pseudomonas syringae* pv. Tomato str. DC300*, and Dickeya dadantii* Ech703 (see Romling and Galperin 2015, Trends Microbiol. 23: 545 PMID: 26077867: Figure 3), which indicates that they might not be universally essential for cellulose production in all bacterial species. For the other operons, e.g. alginate, *pel*, PNAG, and acetylated-cellulose, loci from experimentally characterized species were selected for HMM construction, operon prediction, and downstream multiple sequence alignments and phylogenetic clustering (provided in Supplemental Table 3 – EPS Reference Operons). Multiple sequence alignments of predicted EPS operon associated loci are provided on github (https://github.com/ParkinsonLab/eps_biofilms/).

*• In Figure 2, given the low quality/resolution of the trees presented in figure 2A, I wonder whether this panel is necessary or it could go to the supplemental material.*

We appreciate the reviewer’s comment. We have adjusted Figure 2A to better illustrate the phylogenetic clustering step of our pipeline, indicating in coloured boxes, the delineation of EPS locus clusters and have provided the number of clusters resulting from the use of different cluster quality parameters.

*• Also in Figure 2, it would be of help to keep the colours constant for each EPS operon across panels B and C.*

We have made the correction as suggested.

*• Figure C, phylogenetic distance between loci seems to be sometimes really high > 2. What is the unit represented, SNP/site? Can the authors comment on the robustness of their phylogeny? Bootstrap values are never commented, and it would be of use. Further, do authors believe that with distances over 2, these results are trustworthy? I think this grants a small commentary or caveat section in the discussion*

Distance units are measured in the average number of substitutions per site. The LG model (Le et al. 2008 Philos Trans R Soc Lond B Biol Sci 363: 3965 PMID 18852096) used in estimating evolutionary distance utilizes a matrix mixture approach, incorporating variable rates for distinct regions of protein sequence. Thus certain domains (e.g. membrane embedded) will have greater evolutionary rates and contribute to an increased evolutionary distance overall, despite the conservation of functionally significant residues. During our analyses we also generated multiple sequence alignments for evolutionarily clustered EPS loci sequences and examined their reliability in capturing biologically meaningful information. For example, the selected evolutionary distance threshold of 3.27 average substitutions per site for the cellulose BcsA glycosyltransferase can be compared with the multiple sequence alignment presented in Supplemental Figure 7. It can be clearly seen that even at greater evolutionary distances, the BcsA sequences show differential degrees of conservation associated with the different functional and structural domains; a greater conservation for the glycosyl transferase domain, lower overall conservation for the PilZ domain except for residues critical for c-di-GMP binding, and low conservation of the transmembrane helices of the inner membrane translocon channel.

The comment regarding bootstrap support is well received. We note that bootstrap support has been utilized in some phylogenetic-based clustering approaches (particularly for epidemiological studies of pathogen transmission and evolution e.g. Ragonnet-Cronin et al 2013 BMC Bioinformatics 14: 317 PMID 24191891 and Prosperi et al 2010 PLoS ONE 5: e13619 PMID 21049051). Here we made the decision not to consider bootstrap values in our clustering approach motivated by our desire to provide an automated and agnostic approach for the definition of evolutionarily related clades. Since the phylogenetic distribution, evolutionary selection pressures and sequence sizes of proteins considered in our dataset are variable, at least in the first instance, we would need to consider a semi-automated approach for the selection of bootstrap support parameter for optimal cluster selection.

At the same time, we note that bootstrap support is typically employed in the context of addressing the reliability of phylogenetic tree topologies e.g. Efron et al 1996 PNAS 93: 13429 PMID 8917608). Our approach attempts to define clusters in a topology-agnostic manner by defining clusters based only on their pairwise evolutionary distances. We have incorporated these comments in the methodology section of our manuscript (**Page 33-34, lines 786-793**).

*• L257-26X It would help the reader if the authors would briefly state the function of each protein in the bscABZC operon clearly, as it was done in the next section for the pel operon for instance.*

We have added the additional information to clarify the functional roles of *bcsABCZ* as requested (**Page 12, 273-281**).

*• L347-350 It is not clear whether these two species are known to produce PNAG or this is a putative novel discovery?*

We thank the reviewer for highlighting this important point and have clarified the text to state the novel insights of our findings (**Pages 17-18, lines 400-410**). *E. coli* ETEC H10407 is an enterotoxigenic *E. coli* which elicits diarrhea in humans, is closely related to non-pathogenic commensal and laboratory *E. coli* strains, HS and K-12 (Crossman et al 2010 J Bacteriol 192: 5822 PMID 20802035). Our analysis has identified K-12, a known PNAG producer (Wang et al. 2004 J Bacteriol 186: 2724 PMID 15090514), as possessing a full complement of *pgaABCD,* whereas strain HS is similar to H10407 in lacking the *pgaA* outer-membrane pore locus. These findings are consistent with the evolution of H10407 to a pathogenic lifestyle, where enterotoxins and particularly colonization factors play a critical role for attachment to the intestinal epithelium and enhanced toxicity (Ofek et al. 1990 Infect Immun 58: 3737 PMID 1699898; Madhavan & Sakellaris 2015 Adv Appl Microbiol 90: 155 PMID 25596032).

*• L 472 Is there a word missing?*

We have made the correction (**Page 23, lines 540-541**).

*• L521 there seems to be a problem with the references. It feels that instead of reference #64 it should be #63. Please check this thoroughly throughout the manuscript.*

References have been carefully checked as suggested

*• L631 Authors mention repeatedly to have analysed 1861 bacteria, but table S6 only contains 1388. This is important, specially to evaluate the relative presence of each EPS operon across clades.*

These numbers have now been updated accordingly.

*• L631 I think it would be useful to have some summary statistics concerning the bacteria analysed, concerning number of genomes per bacterial clade, gram –ve vs gram +ve bacteria. I am guessing that the NCBI is very biased towards Gammaproteobacteria.*

We now provide a breakdown of major Taxa as suggested (**Page 30, lines 699-701**)

*• L641 When building HMM profiles, how was the non-redundancy eliminated (> 97% similarity). Which program or settings were used to determine sequence similarity? This should be detailed in the Methods. Wouldn’t protein clustering be a more appropriate method to reduce redundancy?*

We now provide additional details as to how redundancy was eliminated (**Pages 30-31, lines 713-725**).

*• Data availability: I think it would be of use to the community if the HMMs were made available (or if used directly from Pfam: add Pfam link) as well as a simple pipeline for the search to run locally.*

We have made the HMM models used in our prediction pipeline available online from a github repository (<https://github.com/ParkinsonLab/eps_biofilms>) (**Page 31, lines 727-730**).

***Reviewer #3:*** *In this manuscript, the authors state that “a systematic approach for studying the evolution of operon organization is lacking.” They aim to “present a novel method to study the evolution of operons based on phylogenetic clustering of operon-encoded protein families and genomic-proximity network visualizations of operon architectures.”

This is a laudable aim for an important problem.

The authors present methods for analyzing phylogenetic trees to identify clades of functionally similar genes. They then tune their clades using cluster quality measures. This is mostly standard (one might argue about the specific cluster quality measures used, but this is not a major issue). What makes the method more interesting is the joint integration of phylogenetic and genomic-proximity data.

From an algorithmic perspective, there is no question that effective tools and methodologies for these tasks are important, and it is obvious that combining phylogenetic and genomic locus information is essential. As both of these data are graphical, and noisy, the algorithmic challenges are huge. The method that the authors have developed appears to depend on human visual inspection of the operon architectures, and as such, may not be fully automatable for a systematic approach. On the other hand, for such an important task, even small steps towards more effective tools are valuable.

This being said, the paper could benefit from some attention to clarity in the method presentation. This is particularly important as the method is being proposed as a model for others to implement.*

We thank reviewer for their positive and insightful comments.

*For instance, in the subsection titled, “Classification of EPS loci” (lines 671-700) the phylogenetic clustering needs to be clarified. Bootstrap analysis was used, but there is no mention of how (or even if) bootstrap values were incorporated into the method, or even if the final tree analyzed was the consensus tree (or simply the tree constructed from the full multiple sequence alignment). What is meant by “all sequences which share a branch less than the given threshold are assigned to the same evolutionary cluster”? By “branch” do the authors mean tree distance (so that sequences were included in an evolutionary cluster if their tree distance fell within the threshold)?*

We have not used bootstrap support in our clustering step, and have provided a discussion of our rationale in the methodology section of our manuscript (**Pages 33-34, lines 786-793**).

Clustering was performed on the optimized consensus tree. Branch lengths refer to the evolutionary distances parsed from the resulting tree and we have changed our phrasing to reflect this. Only evolutionary distances were considered for clustering, and bootstrap values and tree-topology was not incorporated into the definition of sequence clusters. The reviewer is correct, sequences falling within the evolutionary distance threshold were included into a phylogenetic/evolutionary cluster. We have clarified our clustering procedure in the methodology section of our manuscript (**Page 32, lines 762-766)**.

*As a second example, in the iterative HMM methodology described on page 27, lines 637-644, why did the authors select 20 sequences only to expand the HMM training set? Since HMMs generally need large training sets, this is puzzling.*

As the EPS systems considered in our analysis have been characterized across a variable number of organisms (e.g. cellulose =29, alginate = 5, pel = 4, acet-cellulose = 2, see additional **Supplemental Table 4**), we used the top 20 highest scoring sequence matches to construct our initial HMMs in an attempt to ensure consistency of sampling across EPS systems.

We agree that in general large training sets should be utilized for HMM model generation, but as an important goal of our study was to devise a broadly applicable and agnostic approach for the evolutionary-based clustering of bacterial operons (particularly where a limited number of curated experimentally validated sequences are available), we chose to base our analyses on 20 sequences selected from diverse species (< 97% sequence identity), with the expectation that this would provide an adequate lower-bound on sampling potential amino acid substitutions for sites undergoing random mutation, i.e. not-under functional constraints (**Pages 30-31, lines 713-725**).

Expansion of the training set after the first pass of HMM searches was only possible after genomic-reconstruction of EPS operons, which was used to infer that loci were indeed associated with an EPS operon and not merely structurally related protein families.

*Lastly, although the writing is generally quite good, some editing is needed to fix minor issues in English usage. A few examples are provided:
(a) line 22: “The evolution of these operon-encoded processes is affected by diverse mechanisms such gene duplication, loss, rearrangement, and horizontal transfer.” Insert the word “as” after “such.”*

We have made the correction as suggested (**Page 1, line 24**).

*(b) “gene-families” (line 24) should not be hyphenated.*

This has now been removed.

*(c) line 88: “the inference of biological function based on sequence similarities of genes or proteins are often…” replace “are” with “is”*

We have made the correction as suggested (**Page 5 line 99**).
