# Supplementary material for: A systematic pipeline for classifying bacterial operons reveals the evolutionary landscape of biofilm machineries
Source: PLoS Comput Biol. 2020 Apr 1;16(4):e1007721. doi: 10.1371/journal.pcbi.1007721 (PMC7112194; doi:10.1371/journal.pcbi.1007721)
Supplement: Attachment — Submitted filename: Bundalovic.PlosCB.R1.docx [file pcbi.1007721.s018.docx]

The manuscript entitled « A systematic pipeline for classifying bacterial operons reveals the evolutionary landscape of biofilm machineries » by Bundalovic-Torma and colleagues represent an incredible effort to characterize the composition and evolution of the five major EPS operons known across the bacterial diversity.

I had the chance to review this manuscript in its initial version. The authors have satisfactorily answered/ addressed all the concerns that I raised in my previous review. I believe the manuscript, including the figures, is clearer and has improved significantly. I thus believe this meets the standards of PloS Computational Biology for publication.

Minor comment.

Please revise once again manuscript for minor inconsistencies, for instance, note that the in-file legend of Supplemental Table 8, states it is Supplemental Table 5.
